# Incidence and sociodemographic characteristics of coexisting function-limiting musculoskeletal problems and psychological distress among university students in Sweden: The SUN cohort study

**DOI:** 10.1136/bmjph-2025-003159

**Published:** 2026-08-06

**Authors:** Martina Nanteza, Fred Johansson, Petra Lindfors, Pierre Côté, Eva Skillgate

**Affiliations:** 1 Department of Health Promoting Science, Sophiahemmet University, Stockholm, Sweden; 2 Department of Clinical Neuroscience, Karolinska Institutet, Stockholm, Sweden; 3 Department of Psychology, Stockholm University, Stockholm, Sweden; 4 Institute for Disability and Rehabilitation Research and Faculty of Health Sciences, Ontario Tech University, Oshawa, Ontario, Canada; 5 Unit of Intervention and Implementation Research on Worker Health, Institute of Environmental Medicine, Karolinska Institutet, Stockholm, Sweden

**Keywords:** Anxiety, Depression, Epidemiologic Research Design, Comorbidity, Incidence

## Abstract

**Introduction:**

Musculoskeletal and mental health conditions are common, often coexist and may lead to disability among young adults. We aimed to: (1) estimate the 3-month incidence of coexisting function-limiting musculoskeletal problems and psychological distress among university students in Sweden, (2) estimate recovery from coexisting conditions (resolution of one or both conditions) and (3) determine whether incidence and recovery vary by sociodemographic factors.

**Methods:**

We followed 4262 university students in Sweden (17–65 years; 62% women) over 3 months, assessing function-limiting musculoskeletal problems and psychological distress (moderate or worse depression and/or anxiety) using the Nordic Musculoskeletal Questionnaire and the Depression Anxiety Stress Scales-21. The 3-month incidence of coexisting conditions was estimated among 3642 students without coexisting conditions at baseline. We used risk ratios (RRs) to describe the variation of coexisting conditions across sociodemographic strata, comparing categories within each variable. Recovery at 3 months was estimated among 620 students with both conditions at baseline.

**Results:**

The 3-month incidence of coexisting function-limiting musculoskeletal problems and psychological distress was 8% (n=302, 95% CI 7% to 9%), higher among women (RR: 1.51, 95% CI 1.19 to 1.92), non-Europeans (RR: 1.55, 95% CI 1.17 to 2.06) and students whose parents lacked a university degree (RR: 1.31, 95% CI 1.04 to 1.64). Among students with coexisting conditions at baseline, 42% (n=258, 95% CI 38% to 46%) recovered from at least one condition at 3 months. Recovery did not clearly vary by age group, civil or socioeconomic status, country of origin or parental education.

**Conclusion:**

The 3-month incidence of coexisting function-limiting musculoskeletal problems and psychological distress was 8%, higher in women, non-Europeans and those whose parents had no university degree. Just under half of those with coexisting conditions recovered from at least one condition within 3 months, with no meaningful variation across sociodemographic groups. These findings underscore the need for targeted prevention, integrated student health support and further research.

**WHAT IS ALREADY KNOWN ON THIS TOPIC**

- The coexistence of musculoskeletal and mental health problems is common among young adults, yet little is known about its incidence and recovery specifically in university student populations. Further, it is not known if coexistence varies by sociodemographic factors.

**WHAT THIS STUDY ADDS**

- The incidence of coexisting musculoskeletal problems and psychological distress among university students in Sweden varies by gender, socioeconomic status and country of origin, indicating social inequalities. Most students with coexisting conditions did not recover within 3 months, suggesting a substantial short-term burden.

**HOW THIS STUDY MIGHT AFFECT RESEARCH, PRACTICE OR POLICY**

- These findings underscore the need for targeted, equitable interventions to support students enduring coexisting musculoskeletal and mental health conditions, to potentially facilitate academic achievement, long-term well-being and a resilient future workforce. Further research should identify modifiable risk factors for the coexistence to inform prevention strategies.

## Introduction

 Musculoskeletal (MSK) and mental health (MH) conditions are among the leading global causes of disability,^1^ and often coexist.^2^ In 2021, low back pain, as well as depressive and anxiety disorders, were among the top 10 global contributors to years lived with disability for young people aged 20–24 years.^3^ MSK and MH conditions typically establish their foothold among people before the age of 25,^4,5^ and may impair future educational,^6^ social and economic outcomes.^7,8^ University students bear a significant burden of MSK conditions like neck, shoulder and low back pain,^9^ which have adverse health, social and academic consequences.^10^ Similarly burdensome are MH conditions in university students, resulting in decreased academic performance,^11^ drop out and negative impacts on their careers beyond the university.^6,12^

Models like the biopsychosocial model, the direct causation model and the model of shared or correlated risk factors have been proposed to explain the coexistence of MSK and MH conditions.^13,14^ Moreover, the coexistence has also been attributed to neural and genetic mechanisms.^15–18^ Among American college students, over half with heightened feelings of sadness also reported low back pain, compared with 30% of those without such feelings.^19^ Similarly, among Chinese university students with clinical depression or anxiety, 64% and 65%, respectively, also reported chronic low back pain.^20^ In Portugal, nearly half of university students with temporomandibular symptoms also experienced depression and anxiety.^21^ Among Brazilian students with MSK conditions, 36.2% had depressive symptoms and 50% reported anxiety.^22^

Generally, the coexistence of MSK and MH conditions varies across sociodemographic factors such as age, gender, relationship and socioeconomic status, and country of origin. Age-related patterns are evident; for instance, arthritis tends to coexist with depression or anxiety more frequently in younger adults compared with older ones.^23,24^ Gender disparities are also notable, with women experiencing a greater burden of this coexistence,^25^ likely due to the higher prevalence of both MSK and MH conditions among them.^26,27^ Socioeconomic factors play a critical role, as MSK and MH conditions are more prevalent in less educated and economically deprived individuals.^28–30^ Moreover, in patients with coexistent MSK pain and depression, living with a partner is associated with a lower risk of worsening pain.^31^ The coexistence of MSK and MH conditions is further influenced by ethnicity. Ethnic minorities have a higher occurrence of MSK and MH^32,33^ and have exhibited a stronger association between chronic pain and depression.^34^

There is a paucity of knowledge about the incidence of coexisting function-limiting MSK and MH conditions in university students. This study aimed to determine the 3-month incidence of coexisting function-limiting MSK problems (neck, shoulders, upper back, elbows, hands, lower back, hips, knees and/or, ankles/feet) and psychological distress (PD) (moderate or worse self-reported depression and/or anxiety) among university students in Sweden. Further, the study aimed to determine variations in the incidence across age, gender, civil and socioeconomic status and country of origin. Finally, the recovery from coexisting function-limiting MSK problems and PD after 3 months was assessed.

## Materials and methods

### Study design and sample

This cohort study was based on the Sustainable University Life (SUN) cohort,^35^ comprising a convenience sample of 4262 full-time university students from eight universities in the Stockholm area and Örebro. Students in undergraduate and postgraduate programmes with at least 1 year remaining of their education were enrolled. Participants were recruited at different time points between August 2019 and December 2021 via email invitations containing a link to a web-based baseline survey. Each participant was followed at 3-month intervals for up to one academic year, and the final follow-up point was December 2022. Further details on recruitment procedures and sample size estimation are provided in the published SUN study protocol.^35^

### Patient and public involvement

Focus group interviews with students conducted before the study’s start helped to establish pertinent research questions and guide data collection. Additionally, consultations were carried out with student health services, student unions and the educational offices of universities throughout the study’s design and execution, as well as during the dissemination of the findings.

### Measurements

#### Sociodemographic factors

Self-reported sociodemographic variables were collected at baseline. Age was dichotomised as 25 years and below or above 25 years in the analyses, aligning with the definition of a young adult as someone aged 18–25 years.^36^ Gender was categorised as male, female and other, with ‘other’ including responses ‘other gender identity’ and ‘do not want to identify with any of these identities’. Only male and female participants were included in the analyses pertaining to gender, as the number of participants identifying outside these categories was too small for meaningful analysis. Relationship status was categorised as coupled or single. Country of origin was categorised as Sweden, rest of Europe (originally, answer alternatives were Nordic countries—Norway, Denmark, Finland and Iceland—and Europe excluding the Nordic countries), or outside of Europe. Parents’ highest completed education was categorised as university level or below university level. If at least one parent had a university degree, the parents’ highest education was classified as university level; otherwise, it was classified as below university level. Detailed descriptions of the variables and their measurement are presented in the online supplemental material and the published study protocol.^35^

#### Outcome

Coexistence of MSK problems and PD was defined as the concurrent presence of both conditions at the same assessment, based on participants’ self-reports. Function-limiting MSK problems were assessed using the Nordic Musculoskeletal Questionnaire (NMQ).^37^ The NMQ was selected because it is a standardised instrument with moderate to good reliability in non-clinical and general population-based studies and is widely used to assess MSK symptoms across multiple body regions,^37,38^ enabling identification of overall MSK burden rather than symptoms confined to a single anatomical site. Participants responded ‘Yes’ or ‘No’ to the question: ‘Have you at any time during the last 3 months been prevented from doing your normal activities (eg, work, studies, household work, hobbies) due to trouble with: (1) neck, (2) shoulders, (3) upper back, (4) elbows, (5) hands, (6) lower back, (7) hips, (8) knees and/or, (9) ankles/feet?’ Participants who responded ‘Yes’ for any body area were classified as having function-limiting MSK problems, as this item captures activity limitation due to MSK symptoms within the NMQ framework. The term ‘function-limiting’ was used to align with the WHO conceptualisation of functioning which includes six life domains, for example, activities such as working and studying, and participation in society.^39^ Thus an affirmative answer to this question was considered to reflect functional limitation in the individual students’ ability to perform normal daily and study-related activities due to MSK problems.

PD was defined as the presence of moderate or worse symptoms of depression and/or anxiety (≥7 for depression and ≥6 for anxiety^40^). These symptoms were assessed with the short-form Depression Anxiety Stress Scales (DASS-21).^41^ The DASS-21 consists of 21 items, rated from 0 (‘did not apply to me at all’) to 3 (‘applied to me very much, or most of the time’), with a recall time of 1 week. The items are spread across the three subscales of depression, anxiety and stress, with seven items for each subscale. The total score on each subscale ranges from 0 to 21 and is calculated by summing up the scores of the respective items. The DASS-21 has demonstrated strong construct and convergent validity among Swedish university students.^42^ Furthermore, its subscales have previously exhibited adequate internal consistency^43^; in this study, baseline Cronbach’s alphas were 0.91 and 0.78 for the depression and anxiety subscales, respectively.

### Statistical analysis

The incidence of coexisting MSK problems and PD at the 3-month follow-up was determined among students who had neither or only one of the conditions at baseline. Recovery from coexisting MSK problems and PD was defined as reporting none or only one of the conditions at the 3-month follow-up among students with both conditions at baseline.

The 3-month incidence proportion of coexisting MSK problems and PD was estimated among the 3642 students without both conditions at baseline and presented with 95% CIs. Recovery at 3 months was estimated among the 620 participants who had coexisting MSK problems and PD at baseline. Incidence and recovery were also stratified by sociodemographic characteristics and presented as incidence proportions with 95% CIs. Risk ratios (RRs) with 95% CIs were calculated by dividing the incidence (or recovery) proportion in each exposure category by that in the corresponding reference category. The baseline proportion of coexisting MSK problems and PD at baseline with 95% CI was determined by dividing the number of participants with coexisting conditions by the sample size at baseline (n=4262). Cronbach’s alpha values for the DASS-21 subscales were constructed from the baseline data to determine the internal consistency of the scales in our cohort. All analyses were performed using RStudio V.4.3.2 (2023-10-31 ucrt).

#### Attrition bias

We compared participants lost to follow-up with those retained in the study using a binary variable (yes/no). Baseline sociodemographic characteristics were compared descriptively using cross-tabulations, and crude associations between sociodemographic factors and loss to follow-up were examined using univariable analyses. A multiple logistic regression model was used to assess independent associations between the sociodemographic factors and loss to follow-up, with results presented as ORs with 95% CI.

#### Sensitivity analysis using complete case analysis

In our primary analysis, incidence and recovery were calculated by dividing the number of incident cases at the 3-month follow-up by the number of students at risk at baseline. This led to conservative incidence and recovery estimates when those who were lost to follow-up were included in these analyses. We therefore also conducted complete case analysis (CCA), including only participants with both baseline and 3-month follow-up data. For incidence, 624 participants with missing outcome data were excluded, yielding 3018 complete cases. For recovery, 113 were excluded, resulting in 507 cases. Incidence and recovery proportions were calculated with 95% CI.

## Results

**Figure 1:**
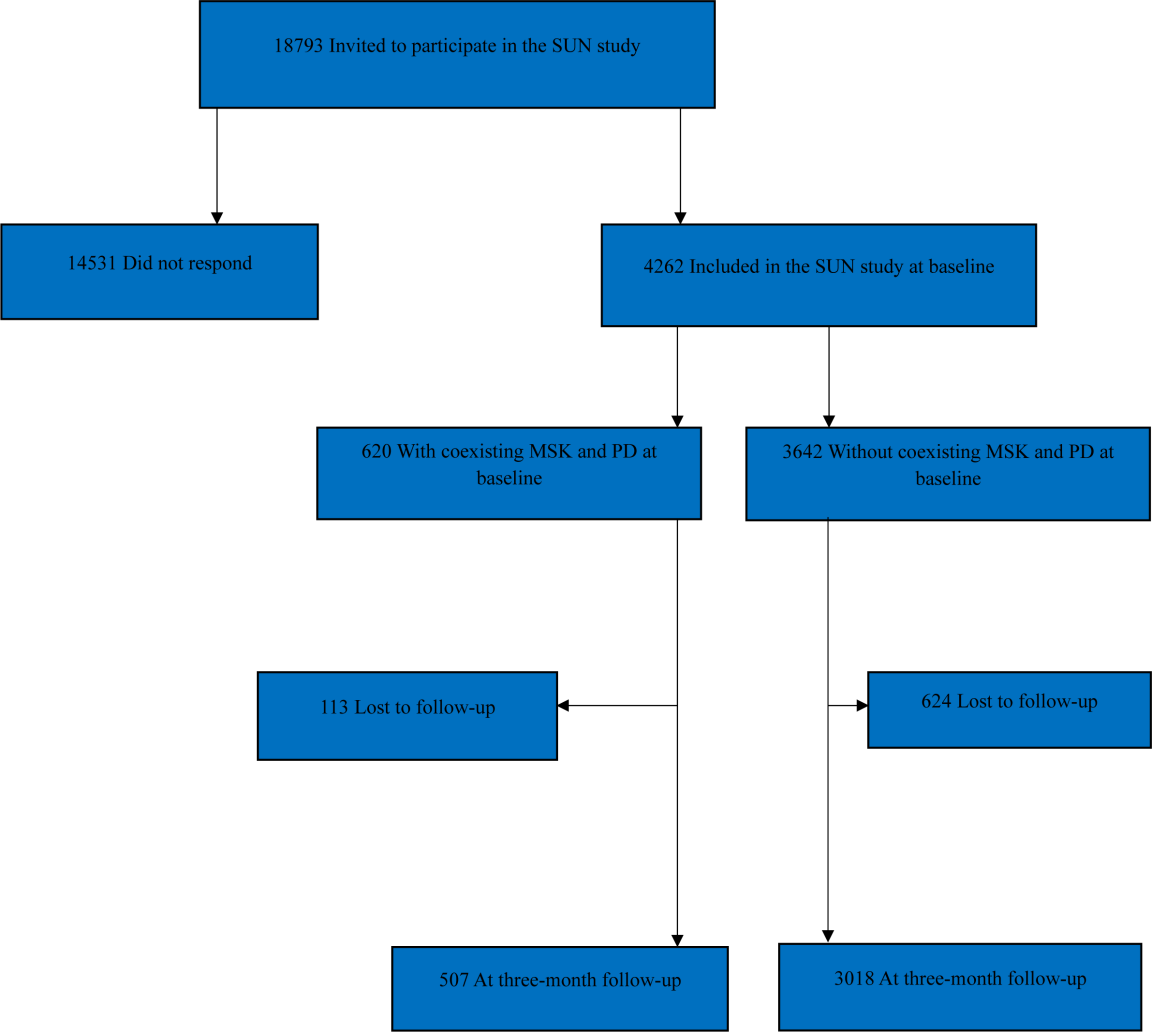
Flowchart of participant inclusion and 3-month follow-up in the SUN study. This flowchart shows the participants’ recruitment, baseline categorisation and follow-up. Of 18 793 invited participants, 4262 enrolled (14 531 did not respond). At baseline, 620 had coexisting musculoskeletal (MSK) problems and psychological distress (PD), while 3642 did not. After 3 months, 507 with coexisting MSK and PD, and 3018 without remained in the study, following 113 and 624 losses to follow-up, respectively.

We invited 18 973 university students to participate in the SUN cohort study, and 4262 agreed. Of these, 737 participants (17%) were lost to follow-up at 3 months (figure 1). The mean age at baseline was 24.6 years (SD=6.1), and most (78%) were of Swedish background, women (62%), aged 25 years and younger (70%) and single (58%). Most were undergraduate students (84%), pursuing education within health sciences (46%) and had at least one university-educated parent (72%). At baseline, 620 (15%) had prevalent coexisting MSK problems and PD. Of the 3642 students without coexisting conditions at baseline, 21% had MSK problems, 23% had PD and 56% had none of the conditions. The descriptive characteristics of the entire study population and of participants based on the coexistence status are presented in table 1. Briefly, sociodemographic characteristics were generally similar between students with and without coexisting conditions at baseline, except for gender. Students with coexisting conditions were more often women (74%) compared with those without coexisting conditions (60%).

**Table 1:** Descriptive baseline characteristics of the participants in the SUN study (n=4262), and according to their musculoskeletal and psychological status at baseline

Characteristics	Entire study population (n=4262)	Coexisting conditions (n=620)	No coexisting conditions (n=3642)
Age,^*^ mean (SD)	24.6 (6.1)	24.7 (5.8)	24.6 (6.1)
Age group, n (%)			
Above 25 years	1276 (30)	194 (31)	1082 (30)
25 years and below	2986 (70)	426 (69)	2560 (70)
Gender,^†^ n (%)			
Male	1592 (37)	157 (25)	1435 (39)
Female	2644 (62)	459 (74)	2185 (60)
Country of origin, n (%)			
Sweden	3337 (78)	473 (76)	2864 (79)
Rest of Europe	401 (9)	58 (9)	343 (9)
Outside Europe	524 (12)	89 (14)	435 (12)
Civil status, n (%)			
Single	2455 (58)	352 (57)	2103 (58)
Coupled	1807 (42)	268 (43)	1593 (42)
Education level, n (%)			
Undergraduate	3593 (84)	526 (85)	3067 (84)
Postgraduate	669 (16)	94 (15)	575 (16)
Education programme, n (%)			
Medical/Health sciences	1965 (46)	302 (49)	1663 (46)
Technical	1170 (42)	254 (41)	1516 (42)
Social sciences	344 (8)	41 (7)	303 (8)
Economics	119 (3)	16 (3)	103 (3)
Other	64 (2)	7 (1)	57 (2)
Parents’ highest completed education, n (%)			
University level	3083 (72)	432 (70)	2651 (73)
Below university level	1179 (28)	188 (30)	991 (27)

Percentages are calculated within each column.

* Age in years.

† 26 participants identifying as neither male nor female were excluded from the analysis.

### Incidence of coexisting MSK problems and PD

The 3-month incidence of coexisting MSK problems and PD was 8% (n=302, 95% CI 7% to 9%) (table 2). Female students were more likely than men to develop coexisting conditions (RR: 1.51, 95% CI 1.19 to 1.92). Single students were not more likely to develop coexisting MSK problems and PD (RR: 1.17, 95% CI 0.94 to 1.47) compared with coupled students. Similarly, non-European students had a higher incidence than their Swedish peers (RR: 1.55, 95% CI 1.17 to 2.06). Finally, the incidence of coexisting MSK problems and PD was higher among students whose parents had no university degree than those with at least one parent with a university degree (RR: 1.31, 95% CI 1.04 to 1.64).

**Table 2:** Incidence of coexisting MSK problems and PD stratified by sociodemographic characteristics at the 3-month follow-up (n=3642)^*^

Categories	No coexisting conditions (n=2716) n (%)	Coexisting conditions (n=302) n (% [95% CI])	Risk ratio [95% CI]
Total	2716 (74)	302 (8 [7 to 9])	–
Age group			
Above 25 years	856 (79)	89 (8 [7 to 10])	1.00
25 years and below	1860 (73)	213 (8 [7 to 10])	1.01 [0.79 to 1.28]
Gender^†^			
Male	1063 (74)	90 (6 [5 to 8])	1.00
Female	1638 (75)	207 (10 [8 to 11])	1.51 [1.19 to 1.92]
Civil status			
Coupled	1206 (78)	116 (8 [6 to 9])	1.00
Single	1510 (72)	186 (9 [8 to 10])	1.17 [0.94 to 1.47]
Country of origin			
Sweden	2210 (77)	221 (8 [7 to 9])	1.00
Rest of Europe	236 (69)	29 (9 [6 to 12])	1.09 [0.76 to 1.59]
Outside Europe	270 (62)	52 (12 [9 to 15])	1.55 [1.17 to 2.06]
Parents’ highest completed education			
University level	1998 (75)	203 (8 [7 to 9])	1.00
Below university level	718 (73)	99 (10 [8 to 12])	1.31 [1.04 to 1.64]

Counts reflect participants with observed 3-month outcome status (analytic sample n=3018).

Percentages and risk ratios are calculated using the baseline population at risk within each stratum as the denominator; participants lost to follow-up contribute to the denominator but not to the numerator.

* Baseline population without coexisting MSK problems and PD=3642; lost to follow-up from baseline=624.

† 26 participants identifying as neither male nor female were excluded from the analysis.

MSK, musculoskeletal; PD, psychological distress.

### Recovery from coexisting MSK problems and PD

Of the 620 students with coexisting MSK problems and PD at baseline (table 3), 42% (n=258, 95% CI 38% to 46%) recovered from at least one condition within 3 months. Recovery was similar across sociodemographic groups. Older students had slightly higher recovery compared with younger students (RR: 1.13, 95% CI 0.92 to 1.39), while women had lower recovery compared with men (RR: 0.88, 95% CI 0.72 to 1.08). Similarly, single students showed slightly higher recovery than coupled students (RR: 1.06, 95% CI 0.88 to 1.28). Students from outside Europe (RR: 0.98, 95% CI 0.75 to 1.28) and those with parents without a university degree (RR: 0.96, 95% CI 0.78 to 1.18) showed marginal differences in recovery compared with Swedish students and those with parents with a university degree, respectively. However, the wide CIs indicate considerable uncertainty around these estimates. Among those with comorbidity at baseline, 11% (n=69) had only MSK problems, 19% (n=119) had only PD and 11% (n=70) had fully recovered at the 3-month follow-up.

**Table 3:** The incidence of recovery from coexisting MSK problems and PD at 3 months, stratified by sociodemographic characteristics (n=620)^*^

Categories	Not recovered (n=249) n (%)	Recovered (n=258) n (% [95% CI])	Risk ratio [95% CI]
Total	249 (40)	258 (42 [38 to 46])	
Age group			
Above 25 years	90 (46)	74 (38 [31 to 45])	1.00
25 years and below	159 (37)	184 (43 [38 to 48])	1.13 [0.92 to 1.39]
Gender^†^			
Male	49 (31)	72 (46 [38 to 54])	1.00
Female	199 (43)	185 (40 [36 to 45])	0.88 [0.72 to 1.08]
Relationship status			
Coupled	120 (45)	108 (40 [34 to 46])	1.00
Single	129 (37)	150 (43 [37 to 48])	1.06 [0.88 to 1.28]
Country of origin			
Sweden	194 (41)	201 (43 [38 to 47])	1.00
Rest of Europe	25 (43)	20 (35 [23 to 48])	0.81 [0.56 to 1.18]
Outside Europe	30 (34)	37 (42 [31 to 53])	0.98 [0.75 to 1.28]
Parents’ highest completed education			
University level	153 (39)	165 (43 [38 to 48])	1.00
Below university level	96 (41)	93 (40 [34 to 47])	0.96 [0.78 to 1.18]

Lost to follow-up from baseline=113; analytic sample at follow-up=507 (620−113).

Counts reflect participants with observed 3-month outcome status (n=507).

Percentages and risk ratios are calculated using the baseline population with coexisting problems within each stratum as the denominator (n=620). Participants lost to follow-up contribute to the denominator but not to the numerator.

* Baseline population with coexisting conditions=620.

† Participants identifying as neither male nor female were excluded from the analysis.

MSK, musculoskeletal; PD, psychological distress.

### Loss to follow-up

In this study, 737 participants of the entire study population were lost to follow-up by 3 months. Participants lost to follow-up were more often younger, men, single and born outside Sweden, while parents’ education was similar between groups (online supplemental table 1). Crude analyses showed similar patterns (online supplemental table 2). In a multiple logistic regression model (online supplemental table 3), all sociodemographic factors except for parents’ highest education were associated with being lost to follow-up. The OR of being lost to follow-up at 3 months was 1.48 (95% CI 1.21 to 1.81) for younger participants, 0.79 (95% CI 0.67 to 0.93) for women, 1.19 (95% CI 1.00 to 1.43) for single participants, 1.87 (95% CI 1.49 to 2.33) for non-Europeans and 1.59 (95% CI 1.23 to 2.05) for non-Swedish Europeans.

### Sensitivity analysis with CCA

Among the 3018 participants with complete follow-up data, the incidence of coexisting conditions was 10% (95% CI 9% to 11%). Of the 507 participants with complete recovery data, 50% (95% CI 46% to 54%) had recovered from at least one of the coexisting conditions.

## Discussion

This cohort study aimed to determine the 3-month incidence of coexisting function-limiting MSK problems and PD among university students in Sweden, to examine variation in incidence across sociodemographic factors and to assess recovery after 3 months. At baseline, 15% of students had coexisting conditions. Among those without coexisting conditions at baseline, 8% had developed such conditions by 3 months, while 42% with baseline coexisting conditions had recovered from at least one condition by 3 months. The incidence was higher among women, non-European students and students whose parents lacked a university degree. However, we found no clear differences in recovery after 3 months across sociodemographic factors. Our findings show that MSK problems and PD commonly coexist among university students and that most affected students do not recover from any of the conditions over 3 months.

To our knowledge, this is the first longitudinal study to estimate the incidence of coexisting function-limiting MSK problems and PD among university students. Our result: incidence of 8%, with a 51% higher risk among female students, is, however, similar to prior general population estimates. The Stockholm Public Health Cohort reported a 5-year cumulative incidence of comorbid MSK problems and PD of 11% for women and 4% for men.^44^ This gender difference, with women more affected by MSK and MH problems, has been attributed to empirically demonstrated factors like hormonal differences^45^ and greater vulnerability.^46^ Additionally, hypothesised factors contributing to this disparity include societal inequalities^47^ and women’s higher healthcare use and self-reporting.^48^ Further, we found that students whose parents had no university degree had a 31% higher incidence of the coexistence of MSK problems and PD. Parental education is a commonly used proxy for socioeconomic status and is known to be associated with children’s health outcomes.^49^ Viewing parental education as an indicator of socioeconomic status, our results are largely consistent with previous research associating socioeconomic deprivation with multimorbidity in general.^50^ Thomson *et al*^30^ proposed a possible explanation for this association, suggesting that both the burden of the primary illness and economic deprivation may contribute to the development of comorbid conditions.

We found that students of non-European descent had a 55% higher incidence of coexisting MSK problems and PD than their Swedish counterparts. These results are consistent with previous research highlighting racial disparities in the comorbidity of MSK and MH problems.^51^ The greater disease burden among ethnic minorities has been linked to poorer health and low socioeconomic status.^33^ We found no age differences in the incidence of coexisting MSK problems and PD among university students. However, most students categorised as older than 25 years in our sample were still below 35 years of age. We also observed no differences in incidence by civil status, despite evidence that higher social support is associated with better mental and MSK health.^52,53^ Our findings suggest that health inequalities among university students are related to gender and other sociodemographic determinants of health. Improving the health and well-being of university students aligns with the United Nations Sustainable Development Goals 3 and 10, which focus on promoting health and reducing inequalities,^54^ and is crucial for fostering resilience, enabling students to contribute positively to societal well-being and play a pivotal role in economic productivity.^55^

The proportion of participants recovering from both MSK problems and PD in our study was less than half that in the Stockholm Public Health Cohort, likely due to the shorter follow-up in our study compared with the Cohort’s 5 years.^44^ However, the overall recovery rate in our study was higher, likely because we defined recovery as the resolution of at least one condition, unlike the Stockholm Cohort, which required the resolution of both comorbid conditions. In addition to being disproportionately affected by coexisting MSK problems and PD, fewer women than men in our study had recovered by 3 months, although there was no significant difference. Similar findings to ours have been reported by Timp *et al*,^56^ who observed that fewer women than men recovered from MH and MSK problems after 3 months. In addition, recovery did not differ by age, socioeconomic status, civil status or one’s country of origin, suggesting that short-term prognosis may be similar across sociodemographic groups.

### Methodological considerations: strengths and limitations

The large cohort size and comprehensive sociodemographic data increased the precision of our estimates and enabled stratified analyses. Still, some estimates had low precision. Furthermore, the DASS-21, which was used to assess symptoms of depression and anxiety, demonstrated acceptable to excellent internal consistency in this study.^57^ Finally, we modified the NMQ scale to assess function-limiting MSK problems over the previous 3 months, to minimise overlapping responses during repeated measurements. This modification is considered to impact minimally on the validity of the NMQ as the shorter recall period potentially decreased the likelihood of misclassification.^58^

The 17% (n=737) loss to follow-up at 3 months might have biased the incidence estimates. Attrition was associated with all included sociodemographic variables except parents’ highest education, suggesting that loss to follow-up did not occur completely at random. Younger, single and non-European participants, who had higher odds of being lost to follow-up, are known from previous research to have higher levels of coexistence between MSK and MH problems. The under-representation of these participants at 3 months likely led to underestimating the overall incidence and the group-specific incidences of the coexistence. However, the CCA yielded an overall incidence of 10% for coexisting conditions, suggesting that missing data had only a minor impact on the primary incidence estimate. Additionally, the estimated proportion of students who recovered might too have been underestimated, as women, who have a higher incidence of coexisting conditions, were less frequently lost to follow-up. Supporting this, findings from the CCA indicated that 50% of participants had recovered from at least one of the coexisting conditions at 3 months, compared with 42% in the primary analysis.

Although follow-up data were available at 3, 6, 9 and 12 months, this study aimed to assess incidence at 3 months. This decision was based on discussions with experienced clinicians in the research group, and the interest is studying short-term fluctuations. Furthermore, higher attrition at later follow-up time-points risked introducing worse attrition bias, where the remaining sample would no longer represent the original population. However, the short follow-up may not capture all incident cases or full resolution of MSK problems and PD.

An important limitation relates to the fact that the recall periods varied between measures of MSK problems and PD (3 months and 1 week, respectively). If there was no overlap in symptom periods, this may have led to misclassification and potentially resulted in overestimation or underestimation of the incidence of coexisting problems and recovery. However, comorbidity does not require conditions to be measured at exactly the same time points.^14^ Furthermore, the 3-month recall period most likely captured persistent MSK symptoms. Also, the DASS-21, although assessing symptoms over the past 7 days, has been found to reflect relatively stable PD^59^ that may coexist with longer-lasting MSK symptoms.

The low response rate of 22% among students invited into the study may have resulted in an underestimation of the baseline proportion of coexisting conditions, as healthier individuals are more likely to participate in surveys.^60^ This potentially compromised the validity of the proportion estimates, limiting their generalisability. Furthermore, the convenience sampling of predominantly medical and technical students restricts the sample’s representativeness of the university population in Sweden.^61^

## Conclusion

The 3-month incidence of coexisting function-limiting MSK problems and PD was 8% among university students in Sweden, higher in women, non-Europeans and those without university-educated parents. Just under half of the students with coexisting conditions at baseline recovered from at least one of them after 3 months, with no variation in recovery rates across sociodemographic groups. These findings highlight the need for targeted prevention and integrated student health support, particularly for vulnerable groups, and for future research on long-term outcomes and interventions.

## Supplementary material

10.1136/bmjph-2025-003159 online supplemental file 1

10.1136/bmjph-2025-003159 online supplemental file 2

## Data Availability

All data relevant to the study are included in the article or uploaded as supplementary information.

## References

[1] Ferrari AJ, Santomauro DF, Aali A (2024). Global incidence, prevalence, years lived with disability (YLDs), disability-adjusted life-years (DALYs), and healthy life expectancy (HALE) for 371 diseases and injuries in 204 countries and territories and 811 subnational locations, 1990–2021: a systematic analysis for the Global Burden of Disease Study 2021. Lancet.

[2] Bair MJ, Robinson RL, Katon W (2003). Depression and pain comorbidity: a literature review. Arch Intern Med.

[3] Institute for Health Metrics and Evaluation (IHME) (2021). 2021 global burden of disease (GBD) compare data visualization. https://vizhub.healthdata.org/gbd-results/?params=gbd-api-2021-public/7c20dd1ebdab1862f20e67bd67ccb85e.

[4] Kessler RC, Berglund P, Demler O (2005). Lifetime prevalence and age-of-onset distributions of DSM-IV disorders in the National Comorbidity Survey Replication. Arch Gen Psychiatry.

[5] Ochsmann EB, Escobar Pinzón CL, Letzel S (2010). Prevalence of diagnosis and direct treatment costs of back disorders in 644,773 children and youths in Germany. BMC Musculoskelet Disord.

[6] Auerbach RP, Alonso J, Axinn WG (2016). Mental disorders among college students in the World Health Organization World Mental Health Surveys. Psychol Med.

[7] Mojtabai R, Stuart EA, Hwang I (2015). Long-term effects of mental disorders on employment in the National Comorbidity Survey ten-year follow-up. Soc Psychiatry Psychiatr Epidemiol.

[8] Goodman A, Joyce R, Smith JP (2011). The long shadow cast by childhood physical and mental problems on adult life. Proc Natl Acad Sci U S A.

[9] Ogunlana MO, Govender P, Oyewole OO (2021). Prevalence and patterns of musculoskeletal pain among undergraduate students of occupational therapy and physiotherapy in a South African university. Hong Kong Physiother J.

[10] Serbic D, Friedrich C, Murray R (2023). Psychological, social and academic functioning in university students with chronic pain: A systematic review. J Am Coll Health.

[11] Keyes CLM, Eisenberg D, Perry GS (2012). The relationship of level of positive mental health with current mental disorders in predicting suicidal behavior and academic impairment in college students. J Am Coll Health.

[12] Rotenstein LS, Ramos MA, Torre M (2016). Prevalence of Depression, Depressive Symptoms, and Suicidal Ideation Among Medical Students: A Systematic Review and Meta-Analysis. JAMA.

[13] da Costa BR, Vieira ER (2010). Risk factors for work-related musculoskeletal disorders: A systematic review of recent longitudinal studies. Am J Ind Med.

[14] Valderas JM, Starfield B, Sibbald B (2009). Defining Comorbidity: Implications for Understanding Health and Health Services. Ann Fam Med.

[15] Hooten WM (2016). Chronic Pain and Mental Health Disorders: Shared Neural Mechanisms, Epidemiology, and Treatment. Mayo Clin Proc.

[16] IsHak WW, Wen RY, Naghdechi L (2018). Pain and Depression: A Systematic Review. Harv Rev Psychiatry.

[17] Goesling J, Clauw DJ, Hassett AL (2013). Pain and depression: an integrative review of neurobiological and psychological factors. Curr Psychiatry Rep.

[18] Lebe M, Hasenbring MI, Schmieder K (2013). Association of serotonin-1A and -2A receptor promoter polymorphisms with depressive symptoms, functional recovery, and pain in patients 6 months after lumbar disc surgery. Pain.

[19] Kennedy C, Kassab O, Gilkey D (2008). Psychosocial factors and low back pain among college students. J Am Coll Health.

[20] Mei Q, Li C, Yin Y (2019). The relationship between the psychological stress of adolescents in school and the prevalence of chronic low back pain: a cross-sectional study in China. Child Adolesc Psychiatry Ment Health.

[21] Minghelli B, Morgado M, Caro T (2014). Association of temporomandibular disorder symptoms with anxiety and depression in Portuguese college students. J Oral Sci.

[22] Soyuer F, Ünalan D, Elmali F (2012). Musculoskeletal complaints of University students and associated physical activity and psychosocial factors. HealthMED.

[23] Guglielmo D, Hootman JM, Boring MA (2018). Symptoms of Anxiety and Depression Among Adults with Arthritis — United States, 2015–2017. MMWR Morb Mortal Wkly Rep.

[24] Keefe FJ, Smith SJ, Buffington ALH (2002). Recent advances and future directions in the biopsychosocial assessment and treatment of arthritis. J Consult Clin Psychol.

[25] Jiménez-Trujillo I, López-de-Andrés A, Del Barrio JL (2019). Gender Differences in the Prevalence and Characteristics of Pain in Spain: Report from a Population-Based Study. Pain Med.

[26] Skillgate E, Pico-Espinosa OJ, Hallqvist J (2017). Healthy lifestyle behavior and risk of long duration troublesome neck pain or low back pain among men and women: results from the Stockholm Public Health Cohort. Clin Epidemiol.

[27] Van de Velde S, Boyd A, Villagut G (2019). Gender differences in common mental disorders: a comparison of social risk factors across four European welfare regimes. Eur J Public Health.

[28] Andorsen OF, Ahmed LA, Emaus N (2017). A prospective cohort study on risk factors of musculoskeletal complaints (pain and/or stiffness) in a general population. The Tromsø study. PLoS ONE.

[29] Callahan LF, Shreffler J, Mielenz T (2008). Arthritis in the family practice setting: associations with education and community poverty. Arthritis Rheum.

[30] Fuller-Thomson E, Nimigon-Young J, Brennenstuhl S (2012). Individuals with fibromyalgia and depression: findings from a nationally representative Canadian survey. Rheumatol Int.

[31] Rambla C, Aragonès E, Pallejà-Millán M (2023). Short and long-term predictors of pain severity and interference in primary care patients with chronic musculoskeletal pain and depression. BMC Musculoskelet Disord.

[32] Scheer J, Costa F, Molinos M (2022). Racial and Ethnic Differences in Outcomes of a 12-Week Digital Rehabilitation Program for Musculoskeletal Pain: Prospective Longitudinal Cohort Study. J Med Internet Res.

[33] Williams ED, Tillin T, Richards M (2015). Depressive symptoms are doubled in older British South Asian and Black Caribbean people compared with Europeans: associations with excess co-morbidity and socioeconomic disadvantage. Psychol Med.

[34] Nicholl BI, Smith DJ, Cullen B (2015). Ethnic differences in the association between depression and chronic pain: cross sectional results from UK Biobank. BMC Fam Pract.

[35] Edlund K, Sundberg T, Johansson F (2022). Sustainable UNiversity Life (SUN) study: protocol for a prospective cohort study of modifiable risk and prognostic factors for mental health problems and musculoskeletal pain among university students. BMJ Open.

[36] Drazdowski TK (2016). A systematic review of the motivations for the non-medical use of prescription drugs in young adults. Drug Alcohol Depend.

[37] Kuorinka I, Jonsson B, Kilbom A (1987). Standardised Nordic questionnaires for the analysis of musculoskeletal symptoms. Appl Ergon.

[38] Dahl AG, Havang S, Hagen K (2022). Reliability of a self-administrated musculoskeletal questionnaire: The fourth Trøndelag health study. Musculoskelet Sci Pract.

[39] World Health Organization (2010). Measuring health and disability: manual for WHO Disability Assessment Schedule (WHODAS 2.0). https://www.who.int/publications/i/item/measuring-health-and-disability-manual-for-who-disability-assessment-schedule-(-whodas-2.0).

[40] Lovibond SH, Lovibond PF (1995). Manual for the depression anxiety stress scales.

[41] Henry JD, Crawford JR (2005). The short-form version of the Depression Anxiety Stress Scales (DASS-21): construct validity and normative data in a large non-clinical sample. Br J Clin Psychol.

[42] Alfonsson S, Wallin E, Maathz P (2017). Factor structure and validity of the Depression, Anxiety and Stress Scale-21 in Swedish translation. J Psychiatr Ment Health Nurs.

[43] Lee B, Kim YE (2022). Validity of the depression, anxiety, and stress scale (DASS-21) in a sample of Korean university students. *Curr Psychol*.

[44] Paanalahti K, Holm LW, Magnusson C (2014). The sex-specific interrelationship between spinal pain and psychological distress across time in the general population. Results from the Stockholm Public Health Study. Spine J.

[45] Kvalheim S, Sandvik L, Winsvold B (2016). Early menarche and chronic widespread musculoskeletal complaints--Results from the HUNT study. Eur J Pain.

[46] Wijnhoven HAH, de Vet HCW, Picavet HSJ (2006). Explaining sex differences in chronic musculoskeletal pain in a general population. Pain.

[47] Patel V (2001). Cultural factors and international epidemiology. Br Med Bull.

[48] Shi P, Yang A, Zhao Q (2021). A Hypothesis of Gender Differences in Self-Reporting Symptom of Depression: Implications to Solve Under-Diagnosis and Under-Treatment of Depression in Males. Front Psychiatry.

[49] Levesque AR, MacDonald S, Berg SA (2021). Assessing the Impact of Changes in Household Socioeconomic Status on the Health of Children and Adolescents: A Systematic Review. Adolesc Res Rev.

[50] Marengoni A, Angleman S, Melis R (2011). Aging with multimorbidity: a systematic review of the literature. Ageing Res Rev.

[51] Kissi A, Vorensky M, Sturgeon JA (2024). Racial Differences in Movement-Related Appraisals and Pain Behaviors Among Adults With Chronic Low Back Pain. J Pain.

[52] Campbell F, Blank L, Cantrell A (2022). Factors that influence mental health of university and college students in the UK: a systematic review. BMC Public Health.

[53] Dunn M, Rushton AB, Mistry J (2024). The biopsychosocial factors associated with development of chronic musculoskeletal pain. An umbrella review and meta-analysis of observational systematic reviews. PLoS One.

[54] United Nations General Assembly (2015). Transforming our world: the 2030 agenda for sustainable development. Report No.: A/RES/70/1.

[55] Khatri P, Duggal HK, Lim WM (2024). Student well-being in higher education: Scale development and validation with implications for management education. Int J Manag Educ.

[56] Timp S, van Foreest N, Roelen C (2024). Gender differences in long term sickness absence. BMC Public Health.

[57] Taber KS (2018). The Use of Cronbach’s Alpha When Developing and Reporting Research Instruments in Science Education. Res Sci Educ.

[58] Althubaiti A (2016). Information bias in health research: definition, pitfalls, and adjustment methods. J Multidiscip Healthc.

[59] Lovibond PF (1998). Long-term stability of depression, anxiety, and stress syndromes. J Abnorm Psychol.

[60] Cheung KL, ten Klooster PM, Smit C (2017). The impact of non-response bias due to sampling in public health studies: A comparison of voluntary versus mandatory recruitment in a Dutch national survey on adolescent health. BMC Public Health.

[61] Bornstein MH, Jager J, Putnick DL (2013). Sampling in Developmental Science: Situations, Shortcomings, Solutions, and Standards. Dev Rev.

[62] European Union (2016). Regulation (EU) 2016/679 of the European parliament and of the council of 27 April 2016: general data protection regulation (GDPR).

