# Supplementary material for: Incidence and sociodemographic characteristics of coexisting function-limiting musculoskeletal problems and psychological distress among university students in Sweden: The SUN cohort study
Source: BMJ Public Health. 2026 Aug 6;4(3):e003159. doi: 10.1136/bmjph-2025-003159 (PMC13448535; doi:10.1136/bmjph-2025-003159)
Supplement: Supplementary file 1 [file bmjph-4-3-s001.pdf]

Categorisation of sociodemographic characteristics.

Age was determined by asking participants to state their age in years and is treated as a categorical variable in this study. **Gender** was assessed by asking, “How do you define your gender identity?” Response options included “Female”, “Male”, “Other”, and “Do not want to identify with either of these identities”. In this study, only the male and female categories were considered. **Parents’ highest education** level was determined by the questions “What is your mother's/father's (parent 1) highest level of education completed?” and “What is your mother's/father's (parent 2) highest level of education completed?” The response categories were “No formal education”, “Less than primary and secondary school (< 9 years)”, “Primary and secondary education (9 years)”, “Upper secondary education (or similar)”, “University (bachelor's or master's)”, “University (doctoral education)” and “Do not know”. For this study, a dichotomous variable was created where the responses “University (bachelor's or master's)” or “University (doctoral education)” on either of the questions were coded as “University level”, and the rest were coded as “Below university.” **Civil status** was assessed by the question “What is your civil status?”, with the response categories as “Unmarried/single”, “Married/partner (living together)”, “Partner (not living together)”, “Divorced/separated” and, “Widowed”. A binary variable in which “Married/partner (living together)” and “Partner (not living together)”, were classified as “Coupled”, and the rest as “Single”, was created. **Country of origin** was assessed by the question “In which country were you born?”, and the responses were “Sweden”, “The Nordic countries (Norway, Denmark, Finland, Iceland)”, “Europe (excluding the Nordic countries)” and “Outside Europe”. A three-level variable was created where “Nordic countries (Norway, Denmark, Finland, Iceland)” and “Europe (excluding the Nordic countries)” were collectively termed as “Rest of Europe”, while the rest were left unaltered. The **education level** was determined by asking “On which level are you studying?” The responses ranged from “Undergraduate studies, semester 1” to “Doctoral studies, semester

7", and were used to create a binary variable with "Undergraduate" and "Post-graduate" as the categories. Participants' **education program** was assessed with the question "In which field of study are you studying?" and the responses were condensed into "medical/health sciences", "technical", "social sciences", "humanities", "economics" and "other."

Supplementary Table 1. Comparison of baseline characteristics between participants lost to follow-up and those retained (N=4262)

**Characteristics**	**Lost to follow-up (N = 737) n (% [95%CI])**	**Not lost to follow-up (N=3525) n (% [95%CI])**
Age group		
Above 25 years	167 (23 [20-26])	1109 (31 [30-33])
25 years and below	570 (77 [74-80])	2416 (69 [67-70])
Gender*		
Male	318 (43 [40-47])	1274 (36 [35-38])
Female	415 (57 [53-60])	2229 (64 [62-65])
Civil status		
Coupled	257 (35 [32-38])	1550 (44 [42-46])
Single	480 (65 [62-68])	1975 (56 [54-58])
Country of origin		
Sweden	511 (69 [66-73])	2826 (80 [79-81])
Rest of Europe	91 (12 [10-15])	310 (9 [8-10])
Outside Europe	135 (18 [16-21])	389 (11 [10-12])
Parents' highest education		
University level	530 (72 [69-75])	2553 (72 [71-74])
Below university level	207 (28 [25-31])	972 (28 [26-29])

Percentages are calculated within each column; totals may not equal 100% due to rounding

*Participants identifying outside of male/female gender were excluded from the analyses with gender

Supplementary Table 2. Cross-tabulation of missingness with sociodemographic characteristics (N=4262;lost to follow-up n = 737)

**Characteristics**	**Loss to follow-up (n= 737) n (% [95%CI])**	**Risk ratio [95% CI]**
Age group		
Above 25 years	167 (13 [11-15])	1.00
25 years and below	570 (19 [18-21])	1.46 [1.24-1.71]
Gender*		
Male	318 (20 [18-22])	1.00
Female	415 (16 [14-17])	0.79 [0.69-0.89]
Civil status		
Coupled	257 (14 [13-16])	1.00
Single	480 (20 [18-21])	1.37 [1.19-1.57]
Country of origin		
Sweden	511 (15 [14-17])	1.00
Rest of Europe	91 (23 [19-27])	1.48 [1.22-1.81]
Outside Europe	135 (26 [22-30])	1.68 [1.43-1.99]
Parents' highest education		
University level	530 (17 [16-19])	1.00
Below university level	207 (18 [15-20])	1.02 [0.88-1.18]

Baseline population = 4262; participants lost to follow-up by three months = 737

Percentages represent the proportion lost to follow-up within each sociodemographic category at baseline

Risk ratios compare the probability of loss to follow-up between categories using the first category as the reference group

*Participants identifying outside of male/female gender were excluded from the analyses with gender

Supplementary Table 3. Multiple logistic regression of loss to follow-up by sociodemographic characteristics

**Characteristics**	**Odds ratio**	**95%CI**
Age group		
Above 25 years	1.00	
25 years and below	1.48	1.21-1.81
Gender *	1.00	
Male		
Female	0.79	0.67-0.93
Civil status		
Coupled	1.00	
Single	1.19	1.00-1.43
Country of origin		
Sweden	1.00	
Rest of Europe	1.59	1.23-2.05
Outside Europe	1.87	1.49-2.33
Parents' highest education		
University level	1.00	
Below university level	1.09	0.90-1.29

Odds ratios represent adjusted associations between baseline sociodemographic characteristics and loss to follow-up at three months from a multiple logistic regression model. The first category of each variable serves as the reference group

*Participants identifying outside of male/female gender were excluded from the analyses with gender
