# Supplementary material for: Incidence and sociodemographic characteristics of coexisting function-limiting musculoskeletal problems and psychological distress among university students in Sweden: The SUN cohort study
Source: BMJ Public Health. 2026 Aug 6;4(3):e003159. doi: 10.1136/bmjph-2025-003159 (PMC13448535; doi:10.1136/bmjph-2025-003159)
Supplement: Supplementary file 2 [file bmjph-4-3-s002.pdf]

BMJ Open

Sustainable UNiversity Life (SUN) study: protocol for a prospective cohort study of modifiable risk and prognostic factors for mental health problems and musculoskeletal pain among university students

Klara Edlund ,[1,2] Tobias Sundberg,[1,2] Fred Johansson,[1,2] Clara Onell,[1,2] Ann Rudman,[3,4] Lena W Holm ,[1,2] Margreth Grotle ,[5] Irene Jensen,[2] Pierre Côté,[6,7] Eva Skillgate[1,2]

To cite: Edlund K, Sundberg T, Johansson F, *et al*. Sustainable UNiversity Life (SUN) study: protocol for a prospective cohort study of modifiable risk and prognostic factors for mental health problems and musculoskeletal pain among university students. *BMJ Open* 2022;**12**:e056489. doi:10.1136/bmjopen-2021-056489

► Prepublication history for this paper is available online. To view these files, please visit the journal online (http://dx.doi.org/10.1136/bmjopen-2021-056489).

Received 18 August 2021
Accepted 03 March 2022

Check for updates

© Author(s) (or their employer(s)) 2022. Re-use permitted under CC BY-NC. No commercial re-use. See rights and permissions. Published by BMJ.

For numbered affiliations see end of article.

Correspondence to
Dr Klara Edlund;
klara.edlund@ki.se

ABSTRACT

Introduction Mental health problems and musculoskeletal pain are common health problems among young adults including students. Little is known about the aetiology and prognosis of these problems in university students. We aim to determine the role of personal, sociodemographic, academic and environmental factors for risk and prognosis of symptoms of depression, anxiety and stress as well as musculoskeletal pain in university students. The constructs that will be studied are based on the biopsychosocial model and psychopathology associated with disabling pain. This model acknowledges illness to consist of interrelated mechanisms categorised into biological, psychological, environmental and social cues.

Methods and analysis This cohort study aims to recruit around 5000 Swedish full-time students. Data will be collected using five online surveys during one academic year. A subgroup (n=1851) of the cohort, recruited before the COVID-19 pandemic, receive weekly text messages with three short questions assessing mood, worry and pain, sent through the web-based platform SMS-track . Statistical analyses will include Kaplan-Meier estimates, Cox regression analyses, multinomial logistic regression analyses and generalised estimating equations. We will assess effect measure modification when relevant and conduct sensitivity analyses to assess the impact of lost to follow-up.

Protocol amendments Due to opportunity and timing of the study, with relevance to the outbreak of the COVID-19 pandemic, this study further aims to address mental health problems, musculoskeletal pain and lifestyle in university students before and during the pandemic.

Ethics and dissemination The Sustainable UNiversity Life study was approved by the Swedish ethics authority (2019-03276; 2020-01449). Results will be disseminated through peer-reviewed research papers, reports, research conferences, student theses and stakeholder communications.

Strengths and limitations of this study

- ► The Sustainable UNiversity Life study will increase the understanding of the aetiology and prognosis of mental health problems and musculoskeletal pain in university students.
- ► The use of valid and reliable instruments and measurement methods and the large number of potential confounders will improve the internal validity of the estimated associations.
- ► Recruitment of participants from large and small universities and colleges with a variety of faculties (medicine, technology, business/economics, social sciences and health sciences) improves external validity.
- ► The study will allow identification of trajectories of mental health problems, musculoskeletal pain and lifestyle before and during the COVID-19 pandemic.
- ► A limitation may be that the majority of the participating students were enrolled at universities in the Stockholm area, and if we fail to include a representative sample, this may threat the external validity mainly of the reports of occurrence and trajectories. Another challenge will be attrition and the risk of selection bias related to that.

Trial registration number NCT04465435.

INTRODUCTION

Mental health problems and musculoskeletal pain

Mental health problems and pain conditions are the highest contributors to global burden of disease. For nearly three decades, low back pain, headache disorders and depressive disorders have prevailed as leading causes of non-fatal health loss.[1] Notably, these

BMJ Open: first published as 10.1136/bmjopen-2021-056489 on 4 April 2022. Downloaded from http://bmjopen.bmj.com/ on February 6, 2025 at Sophiahemmet Hogskila Bibliotek . Protected by copyright, including for uses related to text and data mining, AI training, and similar technologies.

conditions are also among the top 10 causes of the disease burden (quality-adjusted life-years) in adolescents and young adults aged 10–24 years.[2]

There has been an important increase in mental health problems among university students in recent years.[3 4] The onset of mental disorders is most common prior to college and is associated with college drop-out.[5] Similarly, pain is commonly reported among young adults and university students.[2 6] Students who reported low back pain report repercussion on their studies, sleep quality and personal life.[7] Unrelieved pain among students may have adverse impact on their physical and mental health-related quality of life.[8–12] However, the aetiology of comorbid pain and mental health disorders is not well understood. This is significant because death by suicide is a leading cause of mortality worldwide and individuals with chronic pain may be at least twice as likely to report suicidal behaviours or commit suicide.[13] The constructs that will be studied are based on the biopsychosocial model, and psychopathology associated with persistent pain. This model acknowledges illness to consist of interrelated mechanisms categorised into biological, psychological, environmental, and social causes.[14] Along with psychosocial factors being studied, evidence is emerging suggesting persisting pain to be highly correlated with psychopathology, and mostly with depressive disorders, anxiety disorders and substance use disorders, to mention a few.[15–17] The relation between persistent pain and psychopathology is reciprocal and need to be addressed simultaneously. This is the theoretical base of our study aiming to identify and study changes over time in potential risk factors for musculoskeletal pain and mental health problems among students.

In summary

Cohort studies are urgently needed to understand the aetiology of mental health problems and pain conditions in young adults.[18] Studies that help identify modifiable risk and prognostic factors are required to develop health promotion and disease prevention strategies, which ultimately can help reduce the burden of disease in university students. Examples of risk factors suggested in the literature include sleep quality,[19–21] physical activity and a sedentary lifestyle,[22 23] and lifestyle behaviours such as food habits and substance use.[24]

The COVID-19 pandemic and protocol amendments

The outbreak of the COVID-19 pandemic during the spring of 2020, occurred during our ongoing data collection. Protocol amendments were made accordingly in order to assess potential differences in students' health before and during the pandemic. Further, our study lends unique opportunities to address certain questions on how students are affected by COVID-19.

Studies have suggested that factors such as self-quarantine, uncertainty regarding studies, health-related worries and increased loneliness[25–28] may affect university students due to this public health emergency of COVID-19.

STUDY AIM

The overall aim of the Sustainable UNiversity Life (SUN) study is to advance the knowledge about aetiology and prognosis of depression, anxiety, stress and musculoskeletal pain in university students. Our study has three objectives. First, we aim to determine whether possible modifiable factors such as (1) sleep quality, (2) meal patterns, (3) low physical activity/sedentary lifestyle, (4) substance use, (5) study environment, stress, cyber bullying, sexual harassment (6) perfectionism, body image, compulsive training and exercise and loneliness are independent risk factors for the development of incident episodes and unfavourable trajectories of depressive, anxiety and stress symptomatology, as well as musculoskeletal pain in university students. Second, we will investigate whether these factors are independently associated with recovery or worsening of these conditions. Third, we aim to describe the fluctuations of risk and prognostic factors over one academic year, and to determine if such potential risk factors and trajectories vary between men and women.

Long-term follow-up assessments of the cohort are planned within an intended framework of 10 years.

COVID-19 research questions

Research questions related to the COVID-19-pandemic data collection include: Are there any differences in symptoms of depression, anxiety, stress, musculoskeletal pain and lifestyle habits among Swedish university students before and during the COVID-19 outbreak? What factors at baseline are associated with different trajectories of symptoms of depression, anxiety, stress, musculoskeletal pain and lifestyle? What are the trajectories of symptoms of COVID-19 related worry during the pandemic?

METHODS AND ANALYSIS

Study population and selection

Design and source population

The SUN-study is a cohort study of undergraduate- and graduate students (up to master level). Eligible for participation include students, 18 years of age or older, enrolled in full-time educational programmes with at least one academic year before planned graduation. The source population are students at selected universities/colleges in the greater Stockholm area and Örebro attending selected educational programmes. Eight universities, mainly health related with educational programmes in medicine, nursing sciences and life sciences as well as business, technology, and social sciences, will be included. The universities are selected based on geographically feasible locations since meeting with faculty, student health services and students' unions in preparation for data collection require physical presence. The recruitment

BMJ Open: first published as 10.1136/bmjopen-2021-056489 on 4 April 2022. Downloaded from http://bmjopen.bmj.com/ on February 6, 2025 at Sophiahemmet Hogskila Bibliotoket . Protected by copyright, including for uses related to text and data mining, AI training, and similar technologies.

strategy includes physical presence at included universities where in-class presentations about the study will be given. Also, study staff will hand out flyers about the study at public spaces on campus sites, serve coffee and fruits as promotion and answer study-related questions.

Study sample and recruitment process

Inclusion criteria were students at selected universities who were 18 years of age or older, enrolled in a full-time educational programme with at least one academic year before graduation.

A communication plan was developed to achieve a high response rate, both regarding recruiting students to the study and encouraging them to participate at all the follow-up measurements. During the process of recruiting universities and colleges to the study, we are continuously meeting with faculty staff on campus, with student health services, student unions, giving presentations and presenting information, answering questions, handing out flyers and serving coffee to the students on site. We also regularly promote the study on the universities, educational programmes and student unions' social media channels. Recruitment videos were produced with the endorsement from Tim Bergling Foundation. As a way of expressing our appreciation for students' participation, a collaboration has also been established with ACTIC, a Swedish health club chain sponsoring all participants with a 1-month free pass per time they fill out one questionnaire. Prior to the data collection, the universities' educational support offices as well as their student health services are informed about the study and the president's approval is required in order to implement the study. The web-based questionnaire distributed to all eligible students is accessed by a personal link distributed by their personal university email.

Patient and public involvement

There has been an ongoing dialogue with students since the planning of the study started in 2018. This has taken place in class during lectures and via focus group interviews (presented below) as an active part of our communication strategy and to involve students' opinion regarding the content of the survey. Further, discussions are continuously held with student health services and universities' educational offices for collaboration and involvement in the study. Thus, there was an involvement as far as designing and conducting the study as well as in the discussion on the selection of outcome measures and the recruitment of students for participation. The study is continuously disseminated to the public through media, social media and via the universities' webpages.

Data collection

We conducted focus group interviews with two different groups (n=7×2) of undergraduate students from two different universities to plan the design of our web-based questionnaire. As a gesture of appreciation, the students participating in the focus group interviews received a SEK50 (≅ €5; US$5) coffee card at one of the larger chains of coffee houses in Sweden.

Students eligible for the study will mainly be invited to participate through email, with a few exceptions where the universities administer the invitations.

Before inclusion in the study, web-based informed consent is obtained. The baseline survey is estimated to take about 30 min to complete and the follow-up questionnaires about 10–15 min. Recruitment started on August 19, 2019. The four follow-up assessments are distributed every 3 months through out a full academic year. The online platforms used for the data collection is SUNET and Briteback. The baseline questionnaires are hosted online by SUNET Artologik, a secure web-based survey system used in Swedish higher education.[29] The system has previously been used by members of our research group.[30] The follow-up questionnaires are hosted by Briteback, a secure online service that in addition to email also can provide reminders through automatic text messages.[31]

Because the first groups of students were included in August 2019, prior to the outbreak of the COVID-19 pandemic, and because substantial change of study environment due to the restrictions taking place, additional data collection related to the COVID-19 pandemic were added to ensure valid results of our main research questions. This was done with weekly text messages via SMS Track, a web-based software programme designed for research. The technology enables data from a large number of respondents to be gathered every week. The system has previously been used by members of our research group[32] and has also shown to yield high compliance.[33 34] These additional data collections also offer unique opportunities to study potential changes of students' health and lifestyle behaviours during the pandemic.

Technical appendix, statistical code and dataset

The dataset and statistical codes will be available when the data collection is completed.

Potential risk and prognostic factors

Variables potentially associated with increased risk (risk factors), or recovery or recurrence (prognostic factors), of mental health problems or musculoskeletal pain, were identified and selected based on the epidemiological literature as well as on the results of focus group interviews. Well established and validated self-report questionnaires were selected (table 1).

Measurements

Sociodemographic and background questions

Questions in the baseline questionnaire are assessing sociodemographic and background questions (age, gender, level of education, main field of study, sexual orientation and identity, civil status, custody of children, housing, financial status and educational funding, parental educational status, country of origin, overall

BMJ Open: first published as 10.1136/bmjopen-2021-056489 on 4 April 2022. Downloaded from http://bmjopen.bmj.com/ on February 6, 2025 at Sophiahemmet Hogskila Biblioteket . Protected by copyright, including for uses related to text and data mining, AI training, and similar technologies.

Table 1 Measurements in the SUN-study

Variable	Instrument	Baseline	Follow-up
Socio-demographic and background		X	
Sleep	Pittsburgh Sleep Quality Index[38]	X	X
Physical activity and Sedentary behaviour	Single item question for daily sitting time[39]	X	X
Substance use	Alcohol, Smoking, Substance Involvement Screening Test (V.3, Shortened version).[40]	X	X
Study environment Including discrimination	National Survey of Student Engagement, http://nsse.indiana.edu/html/about.cfm	X	X
Cyberbullying	Cyberbullying and Online Aggression Survey Instrument.[41]	X	X
Perfectionism	Frost Multidimensional Perfectionism Scale[42]	X	
Procrastination	Five items based on the Swedish version of the Pure Procrastination Scale[44]		X
Emotion regulation	Compulsive Exercise Test[45]	X	X
Body Image	Body Shape Questionnaire-8-item version[47]	X	
Mental health problems	Depression, Anxiety and Stress Scale 21-item version, short form[35]	X	X
Musculoskeletal pain	Nordic Musculoskeletal Pain Questionnaire[37]	X	X
Social media use	Two items adapted from an original scale about psychological empowerment on social media[56]	X	X
Screen time	Items addressing screen time, including school-work/work and leisure time (computer games, streamed television). Questions developed for this study	X	X
Model Disability Survey	Adapted questions WHO and The World Bank, 2017	X	
Gambling	Problem Gambling Severity Index.[52]	X	
Sexual harassment	Sexual Experience Questionnaire an adaption of selected items.[55] Swedish discrimination law.	X	X
Loneliness<br>Meal frequency	UCLA Three-Item Loneliness Scale (48)<br>One question addressing how many days per week the students are eating their meals (breakfast, snack, lunch, dinner and other meals)	X	X
COVID-19 sub-study weekly text-messages	Three questions about mood, worry and pain intensity.		

health status and whether the student moved to Stockholm to attend the university).

Symptoms of mental health problems are measured by the Depression Anxiety Stress Scale-21 (DASS-21).[35] The original DASS consist of 42 questions but the version used in this study is the now most widely used version with 21 items, assessing symptoms within the last week. The instrument measures three different dimensions of symptoms indicative of mental health problems, Depression, Anxiety and Stress. DASS-21 have reported adequate psychometric properties (test-retest and internal consistency, α 0.81–0.96[35 36]; and has been developed to measure symptoms of depression, anxiety and stress in both clinical and non-clinical populations.

Musculoskeletal pain is evaluated through a modified version of the Nordic Musculoskeletal Questionnaire (NMQ).[37] The questionnaire assessed musculoskeletal symptoms and pain intensity in nine body areas: neck, shoulder, elbow, wrists/hands, upper back, lower back, hips/thighs/buttocks, knees and ankle/feet. The questions were modified to ask about the previous 3 months rather than the previous 12 months as per the original NMQ. The NMQ has been extensively used in the Nordic countries, in more than 100 different projects with a total of more than 50.000 respondents.[37] Reliability tests with test–retest have been conducted as well as validity tests against clinical history with adequate results.[37]

The Pittsburgh Sleep Quality Index

Pittsburgh Sleep Quality Index (PSQI)[38] includes 19 items addressing seven subscales: sleep duration, efficiency, quality, latency, medication use, disturbances and daytime dysfunctions. The PSQI has shown adequate internal consistency, reliability and construct validity. Cronbach's alphas have been reported to 0.80 across groups and correlations between global and component scores have been shown to be moderate to high.[38]

Single item question for daily sitting time

Single item question for daily sitting time (SED-GIH) is a single item question ('How much time do you sit during a normal day, excluding sleep?') with categorical answering

BMJ Open: first published as 10.1136/bmjopen-2021-056489 on 4 April 2022. Downloaded from http://bmjopen.bmj.com/ on February 6, 2025 at Sophiahemmet Hogskila Biblioteket . Protected by copyright, including for uses related to text and data mining, AI training, and similar technologies.

options assessing daily sitting time. The unanchored single item SED-GIH has shown excellent reliability but not adequate validity in investigated populations.[39]

Substance use in the past 3 months will be measured by the Alcohol, Smoking and Substance Involvement Screening Test (ASSIST V.3.0).[40] The ASSIST was developed by the WHO to measure non-medical use of tobacco, alcohol, cannabis, cocaine, stimulants, sedatives, hallucinogens, inhalants, opioids and other drugs. ASSIST has been demonstrated to be a valid screening test for identifying psychoactive substance use in individuals who use a number of substances and have varying degrees of substance use.

Study environment, including questions about discrimination, is measured with questions from the National Survey of Student Engagement; http://nsse.indiana.edu/html/about.cfm), translated and tested for Swedish conditions by the Swedish Higher Education Authority. These questions cover the relevant aspects of students' study environment such as workload, influence, social interactions and feedback. Psychometrics for this scale has, to our knowledge, not been reported.

Cyberbullying is assessed with the Cyberbullying and Online Aggression Survey Instrument, the 'Victimisation' subscale.[41] It measures different types of bullying and harassment online, for example, on social media. Psychometrics for this scale has, to our knowledge, not been reported.

Perfectionism is measured by Frost Multidimensional Perfectionism Scale[42] using the subscales personal standards and concern over mistakes). The subscales have shown acceptable to good internal consistency (Personal standards=0.74; Concern over mistakes=0.86).[43]

Procrastination is measured by five items based on the Swedish version of the Pure Procrastination Scale.[44] The Swedish version of the scales have been shown to have a similar factor structure as the English version, generated good internal consistencies, with Cronbach's alpha ranging between 0.76 and 0.87, and were moderately to highly intercorrelated. Further, the Pure Procrastination Scale, the Irrational Procrastination Scale, and the Susceptibility to Temptation Scale have been reported to be valid and reliable from a psychometric perspectiv.

Emotion regulation by compulsive exercise is measured by the instrument with the same name.[45] Two subscales are included in the study—'Weight control exercise' and 'Avoidance and rule driven behaviour' since these subscales have been reported to have adequate validity (factor analysis) and internal consistency (Weight control exercise: a=0.82, Avoidance and rule driven behaviour: a=0.87).[46] The questions assess behaviours for weight loss and emotion regulation using training.

Body image is measured by the brief version of the Body Shape Questionnaire, the 8-item version (BSQ-8C),[47] based on the full version including 34 items.[48] The questions are reflecting the past 4 weeks. A score <19 indicates no concern with shape, between 19 and 25 mild concern, 26 and 33 moderate concern and >33 marked concern with shape. The eight-item version of BSQ has been found to be sufficiently reliable and valid,[49 50] and the short versions capture the one-factor structure of the full version BSQ.[51] Welch *et al*[47] used the BSQ-8C Swedish version as a stand-alone measurement of body dissatisfaction and found it to show high internal consistency of α=0.94, excellent test–retest properties.

Items addressing screen time regarding social media, schoolwork/work and leisure time (computer games, streamed TV, etc) were measured with questions developed for this study.

The Problem Gambling Severity Index (PGSI)[52] was intended for use in epidemiological research with gamblers across the continuum of risk. The questionnaire has been developed as a part of The Canadian Problem Gambling Index,[53] a questionnaire that measure magnitude and frequency of gambling behaviour as well as individual and social factors relevant to the development of problem gambling. The PGSI has previously been used for epidemiological studies in Canada, Australia, Great Britain, Iceland, Norway and Sweden. The items are scored on a Likert-type scale where the maximum score is 27 (0=no gambling problems; 1–2=Some indication of risk for gambling problems; 3–7=enhanced risk of problem gambling,>8 gambling problems). The PGSI has previously been used in a Swedish cohort study on problem gambling and health.[54]

Sexual harassment is measured using six items based on the Swedish discrimination law. The questions have been extracted and adapted from the Sexual Experience Questionnaire.[55] To our knowledge, no psychometric data have been reported.

Meal frequency is assessed by the question: 'How many days of the week do you eat the following meals?' (breakfast, snack, lunch, snack, dinner, snack, other meals), with the response alternatives 0–7 days/week. The question has been previously used in studies by the research group addressing disturbed eating behaviours as well as reasons for not eating regularly.

Loneliness was measured using the UCLA Three-Item Loneliness Scale.[48] The scale provides a total score ranging from 3 to 9 points. In accordance with previous research, we used a cut-off of ≥6/9 to define loneliness. UCLA three-item Loneliness Scale has acceptable internal consistency (Cronbach's α=0·72 and high correlation (r=0·82)) with the 20 item Revised UCLA Loneliness Scale.

For the part of the cohort that is followed before and during the COVID-19 pandemic, weekly text messages are distributed with three short questions developed by the research group for this purpose. The questions are answered by replying to the text message with a figure, assessing mood, worry and pain by ranking symptoms ranging from well-being to illbeing. The questions are worded as follows:

1. How depressed have you been the past week? Answer with a figure between 0 (not at all) and 10 (worst imaginable).

BMJ Open: first published as 10.1136/bmjopen-2021-056489 on 4 April 2022. Downloaded from http://bmjopen.bmj.com/ on February 6, 2025 at Sophiahemmet Hogskila Biblioteket . Protected by copyright, including for uses related to text and data mining, AI training, and similar technologies.

2. How much pain have you had in the neck and/or back the past week? Answer with a figure between 0 (not at all) and 10 (worst imaginable).
3. How much worry have you felt related to the COVID-19-pandemic the past week? Answer with a figure between 0 (not at all) and 10 (worst imaginable).

Main exposures

Sleep quality, physical activity and sitting time, substance use in the past 3 months (non-medical use), study environment, body image, perfectionism, gambling, compulsive exercise, social media use, cyberbullying, procrastination and loneliness.

Outcomes

Outcomes will be measured with the DASS-21. The DASS-21 includes three subscales designed to measure depression, anxiety and stress symptoms in non-clinical populations with response alternatives on a Likert scale ranging from 0 to 3. Higher scores indicate more severe symptoms. Musculoskeletal pain will be measured with the modified NMQ that cover most potential musculoskeletal pain sights. The questionnaire measure musculoskeletal symptoms, and pain intensity in nine body areas: the neck, shoulder, elbow, wrists/hands, upper back, lower back, hips/thighs/buttocks, knees and ankle/feet. The questions were modified to ask about the previous month rather than the previous 12 months as per the original NMQ. Those reporting no symptoms were assigned a pain score of zero, while those who reported having musculoskeletal symptoms were also asked to report how intense pain was on average over the last month, on a scale from zero, which refers to no complaints, to nine, which refers to the pain 'as bad as it can be'.

Data analysis plan

Definition of trajectories

Trajectories of the outcomes depression, anxiety, stress symptoms, and pain will emerge by latent class growth mixture models (LCGMM) that allows for the identification of multiple underlying trajectories within a defined population. In LCGMM, each trajectory is defined by its own growth parameters (intercept, linear slope), which are assumed to be latent. We will use the Baysian Information Criteria, Bootstrap Likelihood Ratio Test and entropy (measure of uncertainty) to determine whether our four-trajectory hypothesis offers the best fitting solution for the data.

Exploratory analyses

Multinomial logistic regression analyses will be used to determine the associations between each of the exposures and trajectories of outcomes. The associations as ORs and 95% CIs will be reported. Bivariate models to measure the crude associations between the exposures and trajectories will be built. To identify confounders, mediators, and colliders, directed acyclic graphs will be made, in order to select which covariates should be included in the models.

Associations between exposures and incident cases of depression, anxiety, stress symptoms and pain

At baseline, a subcohort of students at risk of developing troublesome symptoms of depression, anxiety and stress as well as pain will be identified. We will use Kaplan-Meier estimates to describe the incidence and discrete time survival analysis to measure the associations between the exposures and the outcome. In all models, the reference category will be the level of exposure hypothesised to be associated with the lowest risk of the outcome. For example, the reference category will be 'not lonely' when analysing the association between loneliness and troublesome depression. HRs and 95% CIs will be used to describe the strength and direction of association. The same approach as described above will be used to identify confounders, mediators, and colliders.

Differences in mental health, lifestyle and pain before and during the COVID-19 pandemic

Generalised estimating equations will be used to estimate mean levels of the outcomes or proportions in each outcome category over time (before and during different time periods of the COVID-19 pandemic) for mental health symptoms, pain, and lifestyle factors. To investigate the role of different exposures on development of the outcomes during the COVID-19 pandemic, models will be fitted including exposure variables with interaction terms between exposures and time, letting the levels of outcome vary by exposure-level over time. Results will be presented as mean or proportion differences between time periods with 95% CI.

Sample size

A number of parameters to be included in multinomial logistic regression models based on distributions of participants across the four hypothesised trajectories have been estimated. The trajectories are: (1) no symptoms, (2) improvement, (3) worsening and (4) persistent. Two different distribution scenarios have been performed. In scenario 1, we hypothesised that 70% have no symptoms, 13% experience worsening, 10% improve and 7% have persistent symptoms. In scenario 2, we hypothesised equal distribution (25%) across trajectories. Based on these assumptions, we estimated that our models could accommodate 32 parameters if we recruit 1000 students (scenario 1) to as many as 234 parameters if we recruit 5000 participants (scenario 2). We estimated that by including approximately 5000 students from invited universities statistical power will be granted.

ETHICS AND DISSEMINATION

All participants provide informed consent to participate in the study, after they have been informed about the purpose and procedure of the study and that it has been approved by the Swedish Ethical Review Authority (reference number: 2019-03276; 2020–01449).

BMJ Open: first published as 10.1136/bmjopen-2021-056489 on 4 April 2022. Downloaded from http://bmjopen.bmj.com/ on February 6, 2025 at Sophiahemmet Hogskila Biblioteket . Protected by copyright, including for uses related to text and data mining, AI training, and similar technologies.

The study is conducted through a web-based self-report survey without any forms of interventions. The survey target potentially sensitive topics such as mental health problems, financial problems and use of alcohol and drugs. The potential risk of participation is negative emotional reactions as a consequence of answering the questions in the survey. Emotional responses among persons at risk for mental health problems may have an increased risk of adverse thoughts being triggered. Student Health Services have been informed about the study and have the capacity to be of service if needed. All students have been informed that participation is voluntary and that they can withdraw from the study at any time. Up to this point, to our knowledge, there have been no indication of our web-based survey causing any pain or discomfort among the participants.

Data are processed in accordance with federal guidelines and laws, and we actively deal with ethical issues that arise during the study. The new regulations for the processing of personal data, the general data protection regulation, is adhered to.

Only the research group will have access to data required to identify the study participants. All results will be presented on group level only and thereby we can ensure that no individual student can be identified.

The dissemination plan is to report results from the study in Swedish reports, journals, international scientific peer reviewed articles and stakeholders.

Trial status

The manuscript reports the protocol (ClinicalTrial.gov PRS) for an ongoing trial for which participants is currently ongoing. The first study participants were included in August 2019 and all included students will be followed for 1 year.

DISCUSSION

The SUN-study will contribute to the knowledge about modifiable risk and prognostic factors for mental health problems and musculoskeletal pain in university students. This new knowledge will contribute to future development of sound evidence-based interventions for prevention of these major global health concerns.

A major strength of the SUN-study is that it will provide a large amount of longitudinal data about university students, a group that have been reported to have high prevalence of these problem areas. The use of mainly valid and reliable methods of measurement limits the risk of misclassification of exposures and outcomes. The information on a large number of potential confounders to consider in the assessment of associations between exposures and outcomes is another strength. Additionally, the sample with students from a variety of small and large universities and colleges as well as different faculties will contribute to ensure external validity. To address the potential risk of attrition in the longitudinal design with repeated measurements, we will encourage participants to fill out all surveys and pay special attention to those who do not, and to potential systematic differences between students taking all surveys and those who do not.

A challenge to the implementation of the SUN-study is the outbreak of the COVID-19 pandemic due to major changes in most aspects of daily lives, also among Swedish students. The Swedish model of restrictions due to the pandemic is widely discussed in international news media, due to Sweden having a different approach than many other countries on how to flatten the curve of the spread of the SARS-CoV-2. On 17 March 2020, all Swedish high schools and universities closed on-campus teaching and extracurricular activities, and mainly remote learning was being conducted. Rules for social distancing as well as recommendations of staying home, refraining from all student activities such as seminars, study groups, student activities, parties, group training at gyms etc were all put on hold. The Swedish restrictions affected university students on most aspects of their daily lives. One potential limitation is that the ongoing COVID-19 pandemic could risk the validity of original research questions in the study, although analyses with repeated measures over time before and during the pandemic is conducted to assess the impact of the COVID-19 outbreak on the sample. These repeated measurements provide unique opportunities to study differences in students' health and lifestyle before and during the pandemic.

Author affiliations
[1]Department of Health Promotion Science, Sophiahemmet University, Stockholm, Sweden
[2]Unit of Intervention Research on Worker Health, Institute of Environmental Medicine, Karolinska Institute, Stockholm, Sweden
[3]Department of Clinical Neuroscience, Karolinska Institutet, Stockholm, Sweden
[4]Department of Health and Welfare, Dalarna University, Falun, Sweden
[5]Department of Physiotherapy, Oslo Metropolitan University, Oslo, Norway
[6]Communication and Research Unit for Musculoskeletal Health (Formi), Oslo University Hospital, Oslo, Norway
[7]Centre for Disability Prevention and Rehabilitation, Faculty of Health Sciences, Ontario Tech University, Oshawa, Ontario, Canada

Acknowledgements We would like to thank ACTIC (health club chain) and Tim Bergling Foundation for their contributions to the project.

Contributors KE is the project director and has contributed to the planning of the study as well as the conception and design and has had the lead position in writing this study protocol. TS is a senior researcher in the project and has contributed to the planning, conception and design of the study including planning and executing the digital survey. FJ and CO are both Ph.D-students who have contributed to the execution of the planned study. They have contributed to the recruitment of the participating universities and administered the work around setting up the study. AR and LH along with Professors MG and IJ have all been senior research advisors with active roles in planning the study, including setting up the design, planning for data analysis and reporting as well as the conception. PC is the co-PI and one of the initiators of the study. He has previously conducted a pilot study on the same topic in Canada which was the starting point for the planning and designing of the present study. ES is the PI, initiator and guarantor. She has led the work on planning, designing and the conception of the study. All authors have contributed to the writing process with input, feedback and critically revising this study protocol. The manuscript was approved by all authors.

Funding This work was supported by the Swedish Research Council for Health, Working Life and Welfare (FORTE), grant number FORTE2018-00402. The project also received financial support from the Public Health Agency of Sweden.

Competing interests None declared.

BMJ Open: first published as 10.1136/bmjopen-2021-056489 on 4 April 2022. Downloaded from http://bmjopen.bmj.com/ on February 6, 2025 at Sophiahemmet Hogskila Bibliotek . Protected by copyright, including for uses related to text and data mining, AI training, and similar technologies.

Patient and public involvement Patients and/or the public were involved in the design, or conduct, or reporting, or dissemination plans of this research. Refer to the Methods section for further details.

Patient consent for publication Not applicable.

Provenance and peer review Not commissioned; externally peer reviewed.

Open access This is an open access article distributed in accordance with the Creative Commons Attribution Non Commercial (CC BY-NC 4.0) license, which permits others to distribute, remix, adapt, build upon this work non-commercially, and license their derivative works on different terms, provided the original work is properly cited, appropriate credit is given, any changes made indicated, and the use is non-commercial. See: http://creativecommons.org/licenses/by-nc/4.0/.

ORCID iDs
Klara Edlund http://orcid.org/0000-0002-2614-5174
Lena W Holm http://orcid.org/0000-0001-6415-8673
Margreth Grotle http://orcid.org/0000-0001-8243-1143

REFERENCES

1 GBD 2017 Disease and Injury Incidence and Prevalence Collaborators. Global, regional, and national incidence, prevalence, and years lived with disability for 354 diseases and injuries for 195 countries and territories, 1990-2017: a systematic analysis for the global burden of disease study 2017. *Lancet* 2018;392:1789–858.
2 Vos T, Lim SS, Abbafati C, *et al*. Global burden of 369 diseases and injuries in 204 countries and territories, 1990–2019: a systematic analysis for the global burden of disease study 2019. *The Lancet* 2020;396:1204–22.
3 Ibrahim AK, Kelly SJ, Adams CE, *et al*. A systematic review of studies of depression prevalence in university students. *J Psychiatr Res* 2013;47:391–400.
4 Lageborn CT, Ljung R, Vaez M, *et al*. Ongoing university studies and the risk of suicide: a register-based nationwide cohort study of 5 million young and middle-aged individuals in Sweden, 1993-2011. *BMJ Open* 2017;7:e014264.
5 Auerbach RP, Alonso J, Axinn WG, *et al*. Mental disorders among college students in the world Health organization world mental health surveys. *Psychol Med* 2016;46:2955–70.
6 Tse MMY, Tang A, Budnick A, *et al*. Pain and pain management among university students: online survey and web-based education. *Cyberpsychol Behav Soc Netw* 2017;20:305–13.
7 Amelot A, Mathon B, Haddad R, *et al*. Low back pain among medical students: a burden and an impact to consider! *Spine* 2019;44:1390–5.
8 Arnow BA, Blasey CM, Lee J, *et al*. Relationships among depression, chronic pain, chronic disabling pain, and medical costs. *Psychiatr Serv* 2009;60:344–50.
9 Dysvik E, Lindstrøm TC, Eikeland O-J, *et al*. Health-Related quality of life and pain beliefs among people suffering from chronic pain. *Pain Manag Nurs* 2004;5:66–74.
10 McCarberg BH, Nicholson BD, Todd KH, *et al*. The impact of pain on quality of life and the unmet needs of pain management: results from pain sufferers and physicians participating in an Internet survey. *Am J Ther* 2008;15:312–20.
11 Merlijn VPBM, Hunfeld JAM, van der Wouden JC, *et al*. Factors related to the quality of life in adolescents with chronic pain. *Clin J Pain* 2006;22:306–15.
12 Moulin DE, Clark AJ, Speechley M, *et al*. Chronic pain in Canada--prevalence, treatment, impact and the role of opioid analgesia. *Pain Res Manag* 2002;7:179–84.
13 Racine M. Chronic pain and suicide risk: a comprehensive review. *Prog Neuropsychopharmacol Biol Psychiatry* 2018;87:269–80.
14 Sullivan MJ, Feuerstein M, Gatchel R, *et al*. Integrating psychosocial and behavioral interventions to achieve optimal rehabilitation outcomes. *J Occup Rehabil* 2005;15:475–89.
15 Hruschak V, Cochran G. Psychosocial and environmental factors in the prognosis of individuals with chronic pain and comorbid mental health. *Soc Work Health Care* 2017;56:573–87.
16 Kamper SJ, Michaleff ZA, Campbell P, *et al*. Back pain, mental health and substance use are associated in adolescents. *J Public Health* 2019;41:487–93.
17 Dersh J, Polatin PB, Gatchel RJ. Chronic pain and psychopathology: research findings and theoretical considerations. *Psychosom Med* 2002;64:773–86.
18 Wykes T, Haro JM, Belli SR, *et al*. Mental health research priorities for Europe. *Lancet Psychiatry* 2015;2:1036–42.
19 Garlow SJ, Rosenberg J, Moore JD, *et al*. Depression, desperation, and suicidal ideation in college students: results from the American foundation for suicide prevention College screening project at Emory University. *Depress Anxiety* 2008;25:482–8.
20 Rasmussen-Barr E, Grooten WJA, Hallqvist J, *et al*. Are job strain and sleep disturbances prognostic factors for low-back pain?A cohort study of a general population of working age in Sweden. *J Rehabil Med* 2017;49:591–7.
21 Hershner S, Chervin R. Causes and consequences of sleepiness among college students. *Nat Sci Sleep* 2014;23:73–84.
22 Annesi JJ, Porter KJ, Hill GM, Grant GM, *et al*. Effects of instructional physical activity courses on overall physical activity and mood in university students. *Res Q Exerc Sport* 2017;88:358–64.
23 Feng Q, Zhang Q-le, Du Y, *et al*. Associations of physical activity, screen time with depression, anxiety and sleep quality among Chinese College freshmen. *PLoS One* 2014;9:e100914.
24 Kwan MY, Arbour-Nicitopoulos KP, Duku E, *et al*. Patterns of multiple health risk-behaviours in university students and their association with mental health: application of latent class analysis. *Health Promot Chronic Dis Prev Can* 2016;36:163–70.
25 Wang X, Hegde S, Son C, *et al*. Investigating mental health of US college students during the COVID-19 pandemic: cross-sectional survey study. *J Med Internet Res* 2020;22:e22817.
26 Elmer T, Mepham K, Stadtfeld C. Students under lockdown: comparisons of students' social networks and mental health before and during the COVID-19 crisis in Switzerland. *PLoS One* 2020;15:e0236337.
27 Cao W, Fang Z, Hou G, *et al*. The psychological impact of the COVID-19 epidemic on college students in China. *Psychiatry Res* 2020;287:112934.
28 Kaparounaki CK, Patsali ME, Mousa D-PV, *et al*. University students' mental health amidst the COVID-19 quarantine in Greece. *Psychiatry Res* 2020;290:113111.
29 SUNET. Skapa enkäter med SUNET Survey [Internet], 2019. Available: https://www.sunet.se/tjanster/survey/ [Accessed 06 Dec 2019].
30 Frögéli E, Rudman A, Gustavsson P. The relationship between task mastery, role clarity, social acceptance, and stress: an intensive longitudinal study with a sample of newly registered nurses. *Int J Nurs Stud* 2019;91:60–9.
31 Briteback, 2019. Available: https://app.briteback.com/ [Accessed 06 Dec 2019].
32 Axén I, Bodin L, Bergström G, *et al*. Clustering patients on the basis of their individual course of low back pain over a six month period. *BMC Musculoskelet Disord* 2011;12:99.
33 Axén I*et al*. Using text messages (SMS) to collect frequent data for evaluation of the six months clinical course of low back pain - scrutiny of a novel method. *European Journal of Pain*.
34 Johansen B, Wedderkopp N. Comparison between data obtained through real-time data capture by SMS and a retrospective telephone interview. *Chiropr Osteopat* 2010;18:10.
35 Lovibond PF, Lovibond SH. The structure of negative emotional states: comparison of the depression anxiety stress scales (DASS) with the Beck depression and anxiety inventories. *Behav Res Ther* 1995;33:335–43.
36 Alfonsson S, Wallin E, Maathz P. Factor structure and validity of the depression, anxiety and stress Scale-21 in Swedish translation. *J Psychiatr Ment Health Nurs* 2017;24:154–62.
37 Kuorinka I, Jonsson B, Kilbom A, *et al*. Standardised Nordic questionnaires for the analysis of musculoskeletal symptoms. *Appl Ergon* 1987;18:233–7.
38 Carpenter JS, Andrykowski MA. Psychometric evaluation of the Pittsburgh sleep quality index. *J Psychosom Res* 1998;45:5–13.
39 Larsson K, Kallings LV, Ekblom Örjan, *et al*. Criterion validity and test-retest reliability of SED-GIH, a single item question for assessment of daily sitting time. *BMC Public Health* 2019;19:17.
40 Humeniuk R, Ali R, Babor TF, *et al*. Validation of the alcohol, smoking and substance involvement screening test (assist). *Addiction* 2008;103:1039–47.
41 Hinduja S, Patchin JW. *Bullying beyond the Schoolyard: preventing and responding to Cyberbullying*. 2nd ed. Thousand Oaks, CA: Sage Publications, 2015.
42 Frost RO, Marten P, Lahart C, *et al*. The dimensions of perfectionism. *Cognit Ther Res* 1990;14:449–68.
43 Bußenius L, Harendza S. The relationship between perfectionism and symptoms of depression in medical school applicants. *BMC Med Educ* 2019;19:370.
44 Rozental A, Forsell E, Svensson A, *et al*. Psychometric evaluation of the Swedish version of the pure procrastination scale, the irrational procrastination scale, and the susceptibility to temptation scale in a clinical population. *BMC Psychol* 2014;2:54.

BMJ Open: first published as 10.1136/bmjopen-2021-056489 on 4 April 2022. Downloaded from http://bmjopen.bmj.com/ on February 6, 2025 at Sophiahemmet Hogskila Bibliotekat . Protected by copyright, including for uses related to text and data mining, AI training, and similar technologies.

45 Taranis L, Touyz S, Meyer C. Disordered eating and exercise: development and preliminary validation of the compulsive exercise test (cet). *Eur Eat Disord Rev* 2011;19:256–68.
46 Plateau CR, Shanmugam V, Duckham RL, *et al*. Use of the compulsive exercise test with athletes: norms and links with eating psychopathology. *J Appl Sport Psychol* 2014;26:287–301.
47 Welch E, Lagerström M, Ghaderi A. Body shape questionnaire: psychometric properties of the short version (BSQ-8C) and norms from the general Swedish population. *Body Image* 2012;9:547–50.
48 Hughes ME, Waite LJ, Hawkley LC, *et al*. A short scale for measuring loneliness in large surveys: results from two population-based studies. *Res Aging* 2004;26:655–72.
49 Cooper PJ, Taylor MJ, Cooper Z, *et al*. The development and validation of the body shape questionnaire. *Int J Eat Disord* 1987;6:485–94.
50 Evans C, Dolan B. Body shape questionnaire: Derivation of shortened "alternate forms". *Int J Eat Disord* 1993;13:315–21.
51 Kling J, Kwakkenbos L, Diedrichs PC, *et al*. Systematic review of body image measures. *Body Image* 2019;30:170–211.
52 Wynne H, Ferris J. *The Canadian Problem Gambling Index: Final Report*. Ottawa: Canadian Centre on Substance Abuse (CCSA), 2001.
53 McCready J, Adlaf E. *Performance and enhancement of the Canadian problem gambling index (CPGI): report and recommendations*. Ottawa: Canadian Centre of Substance Abuse (CCSA), 2006.
54 Statens folkhälsoinstitut. *Spel Om pengar och spelproblem I Sverige 2008/2009. Huvudresultat från SWELOGS befolkningsstudie (Rapport NR. R 2010:23)*. Östersund: Statens folkhälsoinstitut, 2010.
55 Fitzgerald LF, Shullman SL, Bailey N, *et al*. The incidence and dimensions of sexual harassment in academia and the workplace. *J Vocat Behav* 1988;32:152–75.
56 Li Z. Psychological empowerment on social media: who are the empowered users? *Public Relat Rev* 2016;42:49–59.

BMJ Open: first published as 10.1136/bmjopen-2021-056489 on 4 April 2022. Downloaded from http://bmjopen.bmj.com/ on February 6, 2025 at Sophiahemmet Hogskila Biblioteket .
Protected by copyright, including for uses related to text and data mining, AI training, and similar technologies.
